# Genetic Diversity and Population Structure of *Fusarium oxysporum* f. sp. *conglutinans* Race 1 in Northern China Samples

**DOI:** 10.3390/jof8101089

**Published:** 2022-10-16

**Authors:** Jian Ling, Xin Dong, Xingxing Ping, Yan Li, Yuhong Yang, Jianlong Zhao, Xiaofei Lu, Bingyan Xie, Zhenchuan Mao

**Affiliations:** Institute of Vegetables and Flowers, Chinese Academy of Agricultural Sciences, 12 Zhongguancun South Street, Beijing 100081, China

**Keywords:** cabbage fusarium wilt, *Fusarium oxysporum* f. sp. *conglutinans*, genetic diversity, population structure

## Abstract

*Fusarium oxysporum* f. sp. *conglutinans* (FOC), the causal agent of cabbage fusarium wilt, is a serious threat to cabbage production in northern China, and most Chinese FOC isolates were identified as FOC race 1 (FOC1). To better understand the genetic diversity of FOC1 in northern China, we collected FOC isolates from five provinces in northern China and identified them as FOC1 through pathogenicity and race test. To evaluate the genome-level diversity of FOC1, we performed a genome assembly for a FOC1 isolate (FoYQ-1) collected from Yanqing, Beijing, where cabbage fusarium wilt was first reported in China. Using resequencing data of FOC1 isolates, we conducted a genome-wide SNP (single nucleotide polymorphism) analysis to investigate the genetic diversity and population structure of FOC1 isolates in northern China. Our study indicated that Chinese FOC1 can be grouped into four populations and revealed that the genetic diversity of FOC1 were closely associated with geographical locations. Our study further suggests that genetic differentiation occurred when FOC1 spread to the northwest provinces from Beijing Province in China. The FOC1 genetic diversity based on whole-genome SNPs could deepen our understanding of FOC1 variation and provide clues for the control of cabbage fusarium wilt in China.

## 1. Introduction

The fungus *Fusarium oxysporum* (FO) is one of soil-inhabiting fungus causing vascular wilt or root rot in over 120 economically important crops worldwide [1]. According to their pathogenicity to a particular host plant, FO can be divided into more than 100 *formae speciales* (f. sp.), and some *formae speciales* of FO can be further divided into several physiological races [2]. Interestingly, the *forma specialis* of FO are often polyphyletic. Based on sequence similarity for EF-1a gene and ITS sequences, individuals from different *formae speciales* may be more closely related to each other than individuals in the same *forma specialis*, suggesting that pathogenicity to certain plant hosts has independently arisen multiple times from distinct lineages [3]. This most likely resulted from the horizontal transfer of lineage-specific pathogenicity chromosomes, also called accessory chromosomes [4].

*Fusarium oxysporum* f. sp. *conglutinans* (FOC), the causal agent of cabbage Fusarium wilt, is a worldwide threat to cabbage production, resulting in severe economic losses [5]. Based on the pathogenicity with different cabbage genotypes, FOC were referred to two physiological races, race1 (ATCC 52,557, FOC1) and race2 (ATCC 58,385, FOC2), of which FOC1 is a relatively weak pathogenic isolate [6]. FOC1 is the most prevalent race and has been found worldwide [7], while race 2 has been reported only in the United States and Russia until now and can overcome the type-A resistance pattern inherited by a single gene [8,9]. Cabbage is widely planted in China, where more than 900,000 hectares are planted with cabbage every year, accounting for approximately 30% of the world production [10]. Especially in the north of China, cabbage is an important winter vegetable. However, in the past few years, cabbage production in China has suffered from fusarium wilt caused by FOC, resulting in significant production losses [11]. In 2001, FOC was first discovered in Yanqing County, Beijing, China [12], and the damage area has been increasing since then, threatening approximately one-third of the summer and autumn cabbage-growing areas in northern China [13]. Cabbage fusarium wilt has spread rapidly in the planting areas in China since the disease was first reported in China in 2001. Up to now, all the reported FOC isolates in northern China are FOC1 [14].

Some studies showed that with the spread of FOC1 in northern China, the pathogenicity of FOC1 is differentiated, which indicates that FOC1 is differentiated [11,14]. Therefore, it is necessary to analyze the genetic diversity and population structure of FOC1 in China, which could be useful for the development of sustainable disease management strategies for the pathogen. At present, the researches on genetic diversity and population structure of FO mainly uses ITS and EF-1a sequences or PCR-based molecular markers [15,16,17]. The genetic diversity of FOC has also been investigated. Liu et al. analyzed 20 FOC isolates from northern China using ITS and EF-1a sequences and found that FOC1 can be distinguished from other FO, and the genetic distance between FOC1 and FOC2 is greater than that between FOC and other specialized types [14]. However, some studies have shown that ITS and EF-1a sequences cannot accurately reflect the genetic diversity and population structure of FO [18,19]. Genome-wide SNPs or indels are widely used in the study of genetic diversity and population in fungi, which have better resolution of genetic diversity than that of ITS and EF-1a sequences [20]. Halpern et al. found that the phylogenetic tree constructed by SNP is significantly different from the phylogenetic tree based on EF-1a, and the phylogenetic tree constructed by SNP can better reflect the geographical origin of FO on the whole [15].

Thus far, most of the genetic diversity research in FO is to investigate the distribution of different physiological races. Physiological races also differentiate during the process of pathogen spread [21]. Knowledge about the genetic diversity and population structure of specific physiological races is still limited. The genetic diversity based on EF-1a or ITS sequence cannot distinguish the difference among the same physiological race [19]. In this study, we used genome-wide SNPs to analyze the genetic diversity and population structure of 26 FOC1 isolates collected from five provinces in northern China. Our study showed that the phylogenetic tree constructed by genome-wide SNPs was able to show differentiation within FOC1 compared with EF-1a or ITS sequence and revealed that the genetic groups of FOC1 were closely associated with geographical locations of pathogen collection.

## 2. Results

### 2.1. Strains Collection, Identification and Pathogenicity, and Race Test

**Figure 1:**
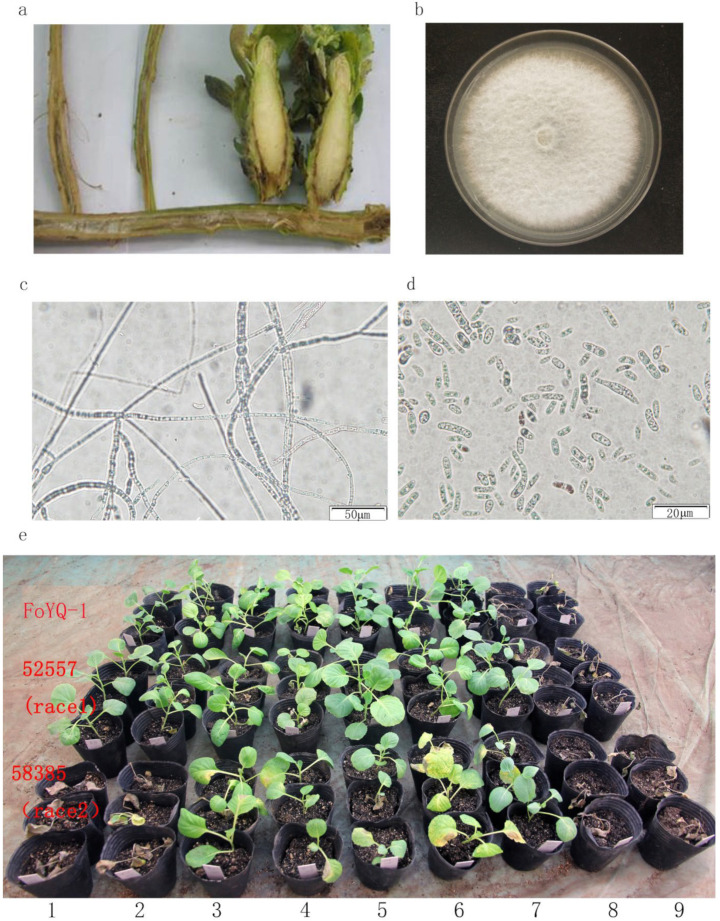
Morphological identification of FOC1 isolates from diseased plants. (**a**) The diseased cabbage plants show typical symptoms of fusarium wilt including stunts, wilts, and vascular necrosis; (**b**) observed colony morphology of FOC1 isolates; (**c**,**d**) microscopic observation on mycelia and conidia of FOC1 isolates; (**e**) different cabbage cultivars were used to FoYQ-1 pathogenicity and race tests. FOC1 race1 (52,557) and FOC race2 (58,385) were used as race test control. The upper two lines were FoYQ-1 pathogenicity test, and the middle two lines and the lower two lines were 52,557 and 58,385 pathogenicity test, respectively. 1 Cultivar “Xiaqiang” was resistant (R) to FOC1 but susceptible (S) to FOC2. 2 Cultivar “Zhonggan18” was R to FOC1 but S to FOC2. 3 Cultivar “Huifeng6” was R to FOC1 and R to FOC2. 4 Cultivar “Lutailang” was R to FOC1 and R to FOC2. 5 Cultivar “Zhenqi” was R to FOC1 and R to FOC2. 6 Cultivar “Huifeng7” was R to FOC1 but S to FOC2. 7 Cultivar “Zhonggan96” was R to FOC1 and R to FOC2. 8 Cultivar “Hanchun1” was S to FOC1 and S to FOC2. 9 Cultivar “Zhonggan21” was S to FOC1 and S to FOC2. The pathogenicity of FoYQ-1 isolates was similar to FOC1.

In this study, we selected three regions in Beijing Province (Changping, Haidian, and Yanqing), two regions in Hebei Province (Baoding and Xintai), two regions in Shanxi Province (Jinzhong and Taiyuan), two regions in Shaanxi Province (Weinan and Xi’an), and two regions in Gansu Province (Dingxi and Yuzhong) for collecting the diseased cabbage, respectively (Table 1). For each region, we selected 2 to 3 fields for collecting the diseased plant with typical symptoms of cabbage fusarium wilt (Figure 1a). Finally, 38 isolates with morphological characteristics similar to those of FOC were obtained from diseased plants. All of the isolates were pathogenic to cabbage cultivar “Zhonggan21” (a widely cultivated susceptible cultivar in northern China) and can be recovered from the inoculated and diseased plants again. The recovered isolates had similar morphological characteristics. Thus, Koch’s postulates were fulfilled.

The morphological analyses of isolated strains were performed to check colony morphology, mycelia, and conidia characteristics. As shown in Figure 1, colony characteristics of the isolates showed a fluffy growth pattern with white mycelial color (Figure 1b). The hyphae were filamentous, colorless, and septate (Figure 1c). The microconidia were hyaline, oval-ellipsoid to cylindrical, erect, or slightly curved with size of 3.4 ± 1.25 in width and 7.2 ± 2.78 in length (Figure 1d). These characteristics agree with the description of FOC [22]. By removing the repeated strains from the same diseased fields, 26 FOC isolates were selected for subsequent study.

We further performed a PCR-based molecular detection on the isolates by using FOC-specific primers. Each of 26 FOC isolates can be amplified a 346 bp FOC-specific DNA fragment, which is the same as that of 52,557 and 58,385, and the 346 bp fragment was not detected in other five FO (Figure S1). As a result, we determined all 26 isolates as FOC.

Pathogenicity and a race test of FOC isolates were carried out on nine cabbage cultivars, including two differential hosts (“XiaQiang” and “Zhonggan18”), and standard FOC1 (52,557) and FOC2 (58,385) were used as control. As shown in Figure 1e, 52,557 were pathogenic to the cultivar “Zhonggan21” and “Hanchun1” but nonpathogenic to the cultivars “XiaQiang” and “Zhonggan18”. Further, 58,385 were pathogenic to all above four cultivars, which is the most significant pathogenic difference between FOC1 and FOC2 (Figure 1e). According to the pathogenicity results of these FOC isolates to nine different cultivars, we found the pathogenicity of these FOC isolates were similar to 52,557. For example, FoYQ-1 was pathogenic to the cultivar “Zhonggan21” and “Hanchun1” but nonpathogenic to the cultivars “XiaQiang” and “Zhonggan18” (Figure 1e). According to the above pathogenicity test, we determined these 26 FOC isolates to be FOC1.

Combined with the above results, we confirmed that all FOC isolates were the FOC1.

### 2.2. FoYQ-1 Genome Assembly and Annotation

**Figure 2:**
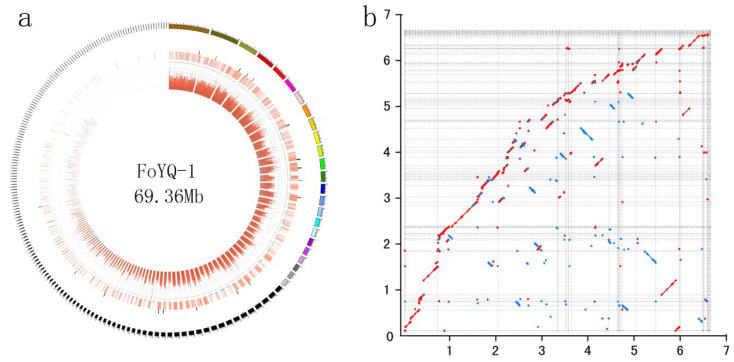
Genome feature of FoYQ-1 and the syntenic relationships with the published chromosome-level FOC genome GCA_014839635.1 (**a**) Genome feature of FoYQ-1. The outermost circle is the contigs. The bar chart from outside to inside in turn is secondary metabolites gene clusters (black), secreted proteins (orange), density of repetitive sequence (blue), and gene density (dark red); (**b**) the syntenic relationships between two genomes. Y-axis represents FoYQ-1 contigs, and x-axis represents GCA_014839635.1 chromosomes. The axis tick values represent genome size (×10 Mb). The red dot or line represents forward matches, and the blue dot or line represents reverse matches between two genomes.

We selected FoYQ-1, which is a FOC1 isolate from Yanqing, Beijing Province, where cabbage fusarium wilt was first reported in China, for genome sequencing. The FoYQ-1 genome size was estimated to be 67 Mb on the basis of the k-mer statistics (Figure S2) from a paired-end library with an insert size of 200 bp. We obtained 5.7 Gb (~85×) of long reads using the PacBio SMART platform for FoYQ-1. The PacBio subreads were assembled into contigs, and a total of 196 contigs were obtained for FoYQ-1, with an N50 of 1.18 Mb (Figure 2a, Table 2). To assess the assembly accuracy, we remapped the raw reads of the paired-end library to the assembled FoYQ-1 genome. The reads covered 95.21% of the genome, with a 97.19% mapping rate and 85× average sequence depth, which implied that the current FoYQ-1 assembly covered almost all unique genomic regions.

A total of 18,319 protein-coding genes were predicted for FoYQ-1. Among the predicted genes, 15,202 (80.51%) genes were functionally annotated. BUSCO assessment of the FoYQ-1 genome showed that 1325 (92.05%) of the gene models were complete (Table 2), suggesting that the assemblies included most of the FoYQ-1 gene space. Compared with the published chromosome-level FOC genome (accession: GCA_014839635.1), two genomes displayed a highly collinear relationship, while some structural variations including inversions and translocations could also be detected (Figure 2b). We identified a total of 163 reversions and 154 translocations (length >10 kb) between two genomes, with the longest inversion of 302.7 kb and longest translocation of 202.4 kb (Table S1), respectively.

### 2.3. Phylogenetic Analyses and Evolutionary Relationships of FOC1

**Figure 3:**
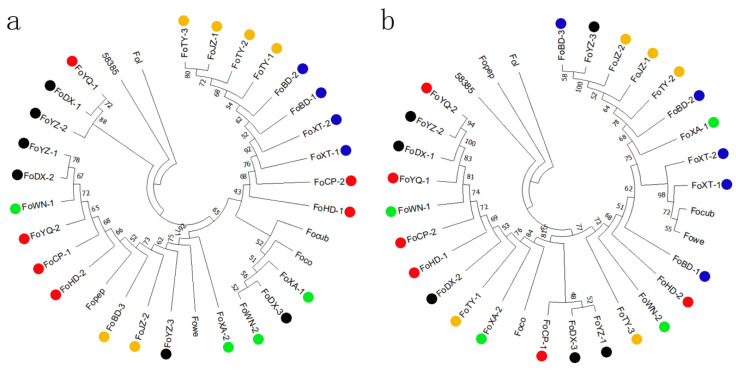
Phylogenetic tree constructed by of EF-1a (**a**) and ITS (**b**) gene sequences using the neighbor-joining (NJ) tree method. Red, blue, yellow, black, and green circle represent the FOC1 isolates collected from Beijing, Hebei, Shanxi, Gansu and Shaanxi Provinces, respectively.

The ITS and the EF-1α sequence of all 26 FOC1 isolates showed the highest identities (99.23% and 99.69%, respectively) with published FO strains sequences when blasted in the NCBI database (Table S2 and S3). These ITS region and EF-1a gene sequences of 26 FOC1 strains and 6 other FO *forma specialis* strains were used to construct phylogenetic trees, respectively (Figure 3a,b). Both phylogenetic trees suggest that FOC2 (58385) and FOC1 strains do not cluster together. We found some other *formae speciales* of FO clustered with FOC1. For example, Focub (host is cucumber) and Fowe (host is melon) clustered with FOC1 isolated collected from Xingtai on ITS phylogenetic tree. Focub and Foco (host is cotton) are clustered with FOC1 isolated from Xi’an on EF-1α tree. It is not clear whether some other *forma specialis* of FO and FOC1 are genetically close, or phylogenetic trees constructed by ITS and EF-1a sequences cannot distinguish the genetic distance between them. The EF-1a phylogenetic trees showed that some FOC1 isolates from the same province were clustered together. For example, FoBD-1, FoBD-2, FoXT-1, and FoXT-2, collected from Hebei Province, were clustered together. However, clustering according to geographical regions was not found in the ITS phylogenetic tree. Our study suggested that EF-1α phylogenetic trees may better reflect the genetic relationship of FOC1 from different geographical regions compared with ITS phylogenetic trees.

### 2.4. Structure Analysis of FOC1

**Figure 4:**
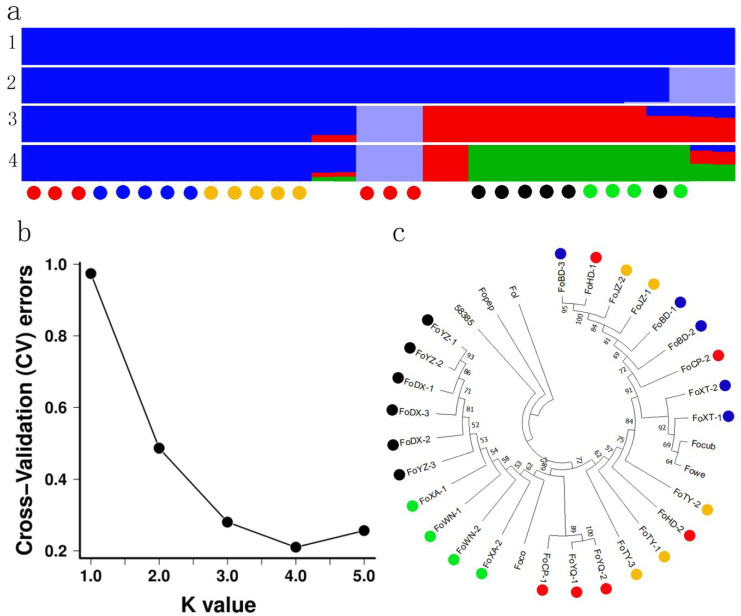
Population structure of FOC1 in northern China. Red, blue, yellow, black, and green circles represent the FOC1 isolates collected from Beijing, Hebei, Shanxi, Gansu, and Shaanxi Provinces, respectively. (**a**) Population structure of 26 FOC1 isolates and other six FO using the admixture software. K is the delta K value for population structure; (**b**) delta K value estimation of sequenced FO; (**c**) neighbor-joining phylogenetic tree constructed by SNP of the 26 FOC1 isolates and the other 6 FO.

The genetic variation and population structure among the FOC1 in northern China were evaluated by resequencing the 26 isolates. We obtained a total of 31.2 Gb, with median depth of 17-fold and 99.21% coverage of the assembled FoYQ-1 genome. After aligning the reads to the FoYQ-1 genome, we identified a total of 264,218 SNPs, with an average of 4.24 SNPs per kb. A total of 13,578 SNPs (5.18%) were located in the coding regions, among which 7098 were non-synonymous SNPs. We used the SNPs to construct a population structure for FOC1 by admixture and explored the population structure of FOC using different delta K (individual ancestry coefficient) values (K value ranged from 1 to 4) (Figure 4a). As shown in Figure 4b, the value of cross-validation error is lowest when delta K is 4, suggesting that four populations (K = 4) can model the data adequately, and other values of K lead to over-fitting. The constructed population structure showed that the FOC1 isolates were divided into four main groups (Figure 4a). The first group (blue) includes FOC1 isolated from Beijing, Hebei, and Shanxi. It is noted that Beijing, Hebei, and Shanxi are neighboring provinces in northern China. The second group (purple) only includes three FOC1 isolated from Beijing. The third group (red) includes FOC2 (58385), and the fourth group (green) includes FOC1 isolates collected from Shaanxi and Gansu Provinces. Shaanxi and Gansu Provinces are neighboring provinces but not close to Beijing and Hebei Provinces. Phylogenetic analysis using SNPs showed that FOC clustered into four clades, which are similar in admixture structure (Figure 4c). One clade contained isolates from Beijing, Hebei, and Shanxi Provinces and is relatively primitive in admixture. The other clade contained FOC1 isolates from Shaanxi and Gansu Provinces. Traditionally, Shaanxi and Gansu were classified as northwest provinces in China, and Beijing, Hebei, and Shanxi Provinces were classified as northern provinces in China. These results imply that genetic differentiation occurred when FOC1 spread to the northwest provinces from Beijing Province in China.

## 3. Discussion

In the control of fusarium wilt, resistant varieties are the most effective measures. Therefore, accurate identification of FO physiological races is essential for disease control [23]. Cabbage is an important vegetable in northern China. However, its production sustains heavy losses from fusarium wilt caused by FOC. FOC has two races, and FOC2 has stronger pathogenicity than FOC1. Previous studies have shown that all FOC isolated in China belong to FOC1, and FOC2 has not been found in China [14]. Researchers have also developed rapid molecular detection methods based on PCR to detect FOC and distinguish FOC from other FO [24]. In this study, we combined the results from morphological analyses, pathogenicity testing, and molecular detection and confirmed that all FOC isolates collected are FOC1, which is the same as the previous study. In China, the cultivars used to control cabbage fusarium wilt, such as “Zhonggan828” and “Zhonggan588”, are highly resist to FOC race 1 but are sensitive to FOC race 2. Because FOC2 has not been found in China, there are few reports on resistance cultivars for FOC race 2 in China. However, as a potential threat for cabbage resistance breeding in China, breeders need to discover and create more resistance resources to FOC race 2 in the future. Although all FOC isolates identified in China are FOC1, these strains showed significant differences in virulence, suggesting that FOC1 has a certain degree of differentiation during its spread in China. Therefore, it is very important to investigate the genetic diversity and population structure of FOC1 in China.

The genetic diversity of FO has also been widely studied. The translation EF-1a and ITS sequences were used in these studies, and many reports showed that compared with ITS sequence, the EF-1a was a suitable genetic marker to distinguish between species. However, many studies have shown that EF-1a cannot fully reflect the genetic structure of FO [17,18,19]. Halpern et al. detected genetic diversity and population structure among 86 diverse FOV isolates and found that the population based on EF-1a genotype is not reflective of FOV isolates’ genetic relatedness, with the exception of race 4 [15]. Liu et al. investigated the genetic diversity of 19 FOC strains and found FOC1 and FOC2 can be distinguished in phylogenetic tree, but it cannot display whether the FOC from different source regions is different [14]. In this study, we found that the phylogenetic tree based on EF-1a and ITS can distinguish FOC1 from other FO and can also distinguish FOC1 from FOC2. This result is consistent with the results of Liu et al. FOC1 from different geographical regions in China did not show significant clustering based on their geographical regions according to the phylogenetic tree constructed by ITS and EF-1a. However, when we used SNPs to construct phylogenetic trees, we found that FOC1 from different geographical regions had a significant differentiation, and FOC1 from different regions usually clustered together, which indicated that FOC1 had a significant differentiation in the process of FOC spread in China. Using SNP and indel to construct the genetic relationship of FO population is more reliable than using ITS and EF-1a. Halpern et al. found that the phylogenetic tree constructed by SNP is significantly different from the phylogenetic tree based on EF-1a, and the phylogenetic tree constructed by SNP can better reflect the geographical origin of FO on the whole [15].

Ma et al. found that the genome of FO is divided into core genome and accessory genome [4]. The accessory genome includes special chromosomes related to the pathogenesis and the *forma specialis* of FO. This chromosome determines the *forma specialis* type of FO. Transferring the special chromosome to different FO can change the *forma specialis* of FO. More and more studies have shown that the physiological race of FO is determined by effectors on the same special chromosome [25,26,27]. Pathogenicity difference between races may be related with secondary metabolism products [28]. Therefore, the difference between FOC1 and FOC2 secondary metabolites need to be studied. In this study, we analyzed the population structure of isolates from five provinces, with the most serious cabbage fusarium wilt in northern China. Our study also has some limitations. First, the number of sampling points in each province is still small. As a result, the number of FOC isolates obtained is small, which may not fully represent the genetic diversity in northern China. In addition, sampling for consecutive years also should be considered to detect the variation of FOC at the same location. We constructed a phylogenetic tree based on genome-wide SNPs. As mentioned above, the specialization type and physiological race of FO are mainly determined by special chromosomes, so the use of SNPs on special chromosomes may more accurately reflect the population structure of FOC1.

In conclusion, we provided genome data of native FOC1 and first used genome-wide SNPs to analyze the genetic diversity and population structure of FOC1 isolates collected from northern China. Our results revealed that the genetic groups of FOC1 were closely associated with geographical locations of pathogen collection and indicate that genetic differentiation occurred when FOC1 spread to the northwest provinces form Beijing Province in China. Our work contributed to the knowledge of FOC1 differentiation in northern China and can provide useful information for cabbage resistance breeding in China.

## 4. Methods

### 4.1. Isolate Collection and Race and Pathogenicity Test

The strains from Fujian of China were a friendly provision from Xiao Rong-feng of the Fujian Academy of Agriculture Science. The strains from Zhejiang of China were a friendly provision from Zhang Zheng-guang of the Zhejiang University. The single-spore cultures of FOC1 were isolated from diseased plants in Beijing, Gansu, Shanxi, Hebei, and Shaanxi Provinces cabbage fields (Table 1).

We selected 11 counties of Beijing, Hebei, Shanxi, Gansu, and Shaanxi Provinces in northern China as sampling points. In each county, diseased plants were collected from 2 to 3 diseased fields in different villages. As a result, a total of 26 sampling regions were selected. FOC isolates were collected from diseased plant tissues according to traditional protocol [29], and the isolated FOC strains were also confirmed by Koch’s postulates. The root-dipping method was used in pathogenicity test according to lv et al. [11]. The cabbage seedlings were cultivated in a greenhouse with a temperature of 28 °C in the day and 20 °C in the night until the third leaf stage. Roots of the seedlings were dipped in the conidial suspension for 15 min, and then, they were planted in plastic pots with sterilized substrate and maintained in the greenhouse with the day temperature of 26 °C–30 °C and night temperature of 22–25 °C. Disease symptoms were measured 8 days after inoculation according to lv et al., and three replicates were performed in the test for each FOC isolates test [11].

A total of nine cabbage cultivars were used as differential hosts to identify races and pathogenicity of the FOC1 isolates. In nine cabbage cultivars, “Xiaqiang”, “Huifeng7”, and “Zhonggan18” are resistant (R) to FOC race 1 (FOC1) but susceptible (S) to FOC race 2 (FOC2). “Huifeng6”, “Lutailang”, “Zhonggan96”, and “Zhenqi” are R to FOC1 and R to FOC2. “Hanchun1” and “Zhonggan21” are S to FOC1 and S to FOC2.

### 4.2. Genome Assembly, Annotation, and Comparative Genomic Analysis

For single-molecule real-time (SMRT) sequencing, only subreads with length more than 500 bp and RQ value higher than 0.75 were retained for future analysis. Canu (v1.6) [30] assembler and DBG2LOC [31] were used for de novo assembly. To improve the assembly result, Quickmerge software [32] was used to merge the two genome assemblies. The genome was first polished by Quiver and followed by three round Pilon [33]. Gene structure and function annotation were carried out according to operation manual [34]. Nucmer of Mummer 3.0 was used for comparative genomic analysis with default parameters.

### 4.3. Molecular Characterization Based on FOC-Specific DNA Fragment and ITS and EF-1a Sequences and Construction of Phylogenetic Trees

Molecular analysis was performed including the FOC-specific, ITS sequence, and EF-1a sequence. The primers for FOC-specific were 5′-TCAATGATAGTGACAAGGGTTT and 5′-AATTTGCTGTGATAGGTGGAT, which can amplify a FOC-specific DNA fragment with size of 346 bp (Table S4). The primers for ITS were 5′-TGTTTCTATATGTAACTTCT and 5′- CAATCAATTTGGGGAACGC, and the primers for EF-1a gene were 5′-AACATGATCACTGGTAAT and 5′-TAAGCAGAAGCCCTTCGC. All the PCR in this study were carried out using 20 µL reaction mixtures, which consist of 1 µL of genomic DNA (100 ng/µL), 2 µL of 10 × PCR Buffer, 0.5 µL of dNTPs (10 mmol), 0.4 µL of EasTaq polymerase (5 U/µL), 0.8 µL of each primer (10 μmol/L) of corresponding primer sets, and added PCR-grade water to the final volume. The parameters for PCR were denatured at 94 °C for 5 min, followed by 35 cycles of denaturing at 94 °C for 30 s, annealing at 63 °C for 30 s, and polymerizing at 72 °C for 0.5–1 min, with a final extension at 72 °C for 10 min.

The nucleotide sequences of the ITS region and EF-1a gene were aligned with ClustalW. Phylogenetic tree was generated by the neighbor-joining (NJ) method with default parameters from the alignment of the nucleotide sequences with MEGA10. Bootstrap analysis with 1000 replications was performed to assess group support.

### 4.4. Resequencing and SNP Calling

For resequencing, a total of 26 isolates were sequenced, and 5 ug DNA for each isolate was used for library construction. The libraries with 350 bp insert size were sequenced on the Illumina HiSeq 2500, generating 150 bp paired-end reads. Raw reads were subjected to removal of adaptors and trimmed of low-quality bases. We obtained a total of 31.2 Gb clean data, with median depth of 17× coverage for each isolate. The clean data of each individual were mapped to the FoYQ-1 genome using BWA (Version 0.6.2). Then, samtools (version 1.1, Genome Research Ltd., Saffron Walden, UK) was used to transform the format and sort the result of BWA. The variations were called using GATK (4.1.1, Broad Institute, Cambridge, MA, USA) according to the GenotypeGVCFs pipeline. Plink software with the parameters -geno 0.2, -maf 0.05 was used to filter the variations and obtain high-quality SNPs and small indels.

### 4.5. Diversity and Structure Analysis

The ADMIXTURE software was used to construct a population structure of FOC1 isolates (with the parameters –C 0.001 –j10 –seed1). A custom R script was used to confirm and plot the optimal population structure.

## Figures and Tables

**Table 1:** FOC1 isolates and other FO used in this study.

	Strains	Scientific Name	Hosts	Sources	Sampling Coordinates
1	FoYZ-1	*F. oxysporum* f. sp. *conglutinans*	Cabbage	Yuzhong, Gansu	35.9131 N/104.1622 E
2	FoYZ-2	*F. oxysporum* f. sp. *conglutinans*	Cabbage	Yuzhong, Gansu	35.9128 N/104.1659 E
3	FoYZ-3	*F. oxysporum* f. sp. *conglutinans*	Cabbage	Yuzhong, Gansu	35.9141 N/104.1573 E
4	FoYQ-1	*F. oxysporum* f. sp. *conglutinans*	Cabbage	Yanqing, Beijing	40.4302 N/115.9757 E
5	FoYQ-2	*F. oxysporum* f. sp. *conglutinans*	Cabbage	Yanqing, Beijing	40.4287 N/115.9782 E
6	FoXT-1	*F. oxysporum* f. sp. *conglutinans*	Cabbage	Xintai, Hebei	37.1977 N/114.6448 E
7	FoXT-2	*F. oxysporum* f. sp. *conglutinans*	Cabbage	Xintai, Hebei	37.1926 N/114.6465 E
8	FoXA-1	*F. oxysporum* f. sp. *conglutinans*	Cabbage	Xi’an, Shaanxi	34.3253 N/109.1893 E
9	FoXA-2	*F. oxysporum* f. sp. *conglutinans*	Cabbage	Xi’an, Shaanxi	34.3280 N/109.1932 E
10	FoWN-1	*F. oxysporum* f. sp. *conglutinans*	Cabbage	Weinan, Shaanxi	34.6500 N/109.6391 E
11	FoWN-2	*F. oxysporum* f. sp. *conglutinans*	Cabbage	Weinan, Shaanxi	34.6493 N/109.6400 E
12	FoTY-1	*F. oxysporum* f. sp. *conglutinans*	Cabbage	Taiyuan, Shanxi	37.9148 N/112.4041 E
13	FoTY-2	*F. oxysporum* f. sp. *conglutinans*	Cabbage	Taiyuan, Shanxi	37.9134 N/112.4090 E
14	FoTY-3	*F. oxysporum* f. sp. *conglutinans*	Cabbage	Taiyuan, Shanxi	37.9184 N/112.4021 E
15	FoJZ-1	*F. oxysporum* f. sp. *conglutinans*	Cabbage	Jinzhong, Shanxi	37.7784 N/112.8786 E
16	FoJZ-2	*F. oxysporum* f. sp. *conglutinans*	Cabbage	Jinzhong, Shanxi	37.7843 N/112.8780 E
17	FoHD-1	*F. oxysporum* f. sp. *conglutinans*	Cabbage	Haidian, Beijing	39.9678 N/116.3387 E
18	FoHD-2	*F. oxysporum* f. sp. *conglutinans*	Cabbage	Haidian, Beijing	39.9672 N/116.3398 E
19	FoDX-1	*F. oxysporum* f. sp. *conglutinans*	Cabbage	Dingxi, Gansu	35.5788 N/104.5490 E
20	FoDX-2	*F. oxysporum* f. sp. *conglutinans*	Cabbage	Dingxi, Gansu	35.5811 N/104.5496 E
21	FoDX-3	*F. oxysporum* f. sp. *conglutinans*	Cabbage	Dingxi, Gansu	35.5811 N/104.5496 E
22	FoCP-1	*F. oxysporum* f. sp. *conglutinans*	Cabbage	Changping, Beijing	40.2432 N/116.3885 E
23	FoCP-2	*F. oxysporum* f. sp. *conglutinans*	Cabbage	Changping, Beijing	40.2432 N/116.3851 E
24	FoBD-1	*F. oxysporum* f. sp. *conglutinans*	Cabbage	Baoding, Hebei	38.8809 N/115.6137 E
25	FoBD-2	*F. oxysporum* f. sp. *conglutinans*	Cabbage	Baoding, Hebei	38.8826 N/115.6102 E
26	FoBD-3	*F. oxysporum* f. sp. *conglutinans*	Cabbage	Baoding, Hebei	38.8797 N/115.6127 E
27	Fopep	*F. oxysporum* f. sp. *capsicum*	Pepper	Fujian, China	-
28	Foco	*F. oxysporum* f. sp. *cowpea*	Cowpea	Fujian, China	-
29	Focub	*F. oxysporum* f. sp. *cucumerinum*	Cucumber	Beijing, China	-
30	Fowe	*F. oxysporum* f. sp. *melonis*	Muskmelon	Fujian, China	-
31	Fol	*F. oxysporum* f. sp. *lycopersici*	Tomato	Zhejiang, China	-
32	58,385	*F. oxysporum* f. sp. *conglutinans*	Cabbage	ATCC	-
33	52,557	*F. oxysporum* f. sp. *conglutinans*	Cabbage	ATCC	-

ATCC, American Type Culture collection.

**Table 2:** Statistics of the FoYQ-1 assembly.

Indicators of Genome Assembly	FoYQ-1
Length of genome assembly (Mb)	69.36
Number of contigs	192
N50 of contigs (Mb)	1.18
Total length of retrotransposons (Mb)	34.41
Number of annotated genes	18,319
Average gen length (bp)	2646
Average CDS length (bp)	1368
Average protein length (bp)	455
Genome completeness (BUSCO)	92.05%

## Data Availability

FoYQ-1 genome data has been deposited to the National Genomics Data Center, under accession number GWHBKCM00000000.

## Supplementary Materials

The following supporting information can be downloaded at: www.mdpi.com/10.3390/jof8101089/s1, Figure S1: PCR identification of FOC using FOC-specific primer; Figure S2: K-mer (25-mer) analysis for estimating the genome size of FoYQ-1; Table S1: The reversions and translocations between two genomes; Table S2: EF-1a sequences of 26 FOC1 isolates blast to NCBI; Table S3: ITS sequences of 26 FOC1 isolates blast to NCBI; Table S4: The sequence of FOC-specific DNA fragment.
