# Supplementary material for: Genetic Diversity and Population Structure of Fusarium oxysporum f. sp. conglutinans Race 1 in Northern China Samples
Source: J Fungi (Basel). 2022 Oct 16;8(10):1089. doi: 10.3390/jof8101089 (PMC9604595; doi:10.3390/jof8101089)
Supplement: Supplementary file 1 [file jof-08-01089-s001.zip › jof-1916606-supplementary.pdf]

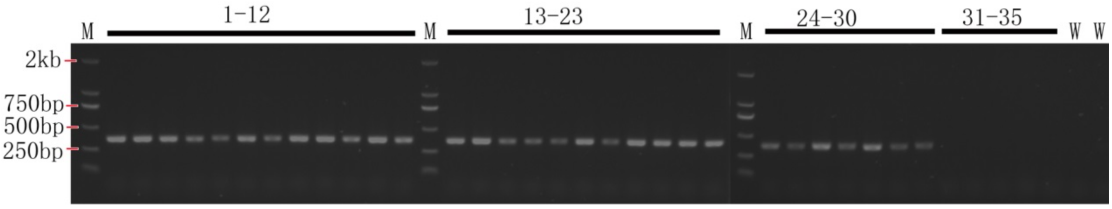

Figure S1. PCR identification of FOC using FOC-specific primer. M，molecular markers of D2000 DNA marker. 1-26, 26 FOC isolates corresponding to the strains listed in table 1 from 1-26. 27-28, FOC race 1(ATCC 52557). 29-30, FOC race 2(ATCC 58385). 31-35, five other FO used as control(31, Fopep. 32, Foco. 33, Focub, 34, Fowe. 35, Fol). W, negative control using PCR-grade water as the template. A 346 bp FOC-specific DNA fragment can be amplified in all FOC isolates, 52557 and 58385.

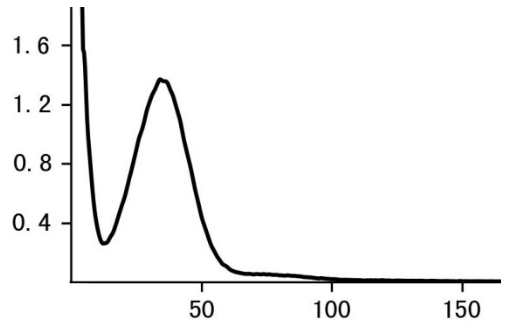

Figure S2. K-mer (25-mer) analysis for estimating the genome size of FoYQ-1. The X-axis is k-mer depth and the Y-axis is the proportion that represents the frequency at that depth. The peak K-mer frequency was 35.

Table S1. The reversions and translocations between two genomes. GCA_014839635.1 used as reference.

reference	begin	end	query	begin	end	matchlength	similarity	type
MU089289.1	228903	531677	tig00000079	925223	622450	302775	99.99	reversion
MU089289.1	4115240	4317690	tig00001019	586344	788793	202451	99.99	translocation
MU089288.1	891536	1024978	tig00001021	196933	330373	133443	99.99	translocation
MU089289.1	1990231	2106395	tig00001034	577685	461504	116165	99.98	reversion
MU089289.1	4024955	4113314	tig00001019	497957	586345	88360	99.96	translocation
JABTBS010000012.1	3447982	3534870	tig00001052	788515	701648	86889	99.79	reversion
MU089289.1	727954	813234	tig00000079	316651	231402	85281	99.96	reversion
JABTBS010000021.1	30065	112197	tig00000173	93526	11394	82133	99.99	reversion
MU089289.1	4317689	4399036	tig00001019	791010	872350	81348	99.95	translocation
JABTBS010000013.1	107005	187360	tig00009036	1651210	1731561	80356	99.99	translocation
MU089289.1	142633	222790	tig00000079	1005382	925223	80158	99.93	reversion
JABTBS010000013.1	24357	88766	tig00001103	177875	113469	64410	99.99	reversion
JABTBS010000006.1	2234768	2297768	tig00000086	1841813	1778813	63001	99.99	reversion
MU089288.1	3723333	3785257	tig00009038	61900	1	61925	99.88	reversion
MU089291.1	9328	64573	tig00001065	19693	74937	55246	99.99	translocation
MU089289.1	673786	727954	tig00000079	415387	361219	54169	99.99	reversion
JABTBS010000038.1	65815	119503	tig00001092	380406	434093	53689	99.99	translocation
MU089289.1	615338	665671	tig00000079	475884	425551	50334	99.99	reversion
MU089288.1	3006020	3056342	tig00001078	28338	78661	50323	99.64	translocation
MU089289.1	566437	610773	tig00000079	520137	475800	44337	99.96	reversion
JABTBS010000015.1	3019473	3060226	tig00001083	869517	828764	40754	99.99	reversion
JABTBS010000014.1	315378	355675	tig00001068	11233	51531	40298	99.78	translocation
MU089289.1	3158008	3196972	tig00001027	1	38961	38965	99.89	translocation
JABTBS010000043.1	62309	99996	tig00000441	3	37686	37688	99.97	translocation
JABTBS010000012.1	3411423	3446054	tig00001052	823173	788516	34632	99.92	reversion
MU089288.1	3662923	3694828	tig00009038	125556	93665	31906	99.96	reversion
JABTBS010000006.1	2201997	2232842	tig00000086	1872657	1841812	30846	100	reversion
JABTBS010000014.1	2553167	2583622	tig00001040	34660	4175	30456	99.89	reversion
JABTBS010000014.1	2585724	2616179	tig00001040	34660	4175	30456	99.89	reversion
JABTBS010000043.1	1	29820	tig00000441	37729	67553	29820	99.98	translocation
MU089289.1	820011	848756	tig00000079	221419	192676	28746	99.99	reversion
MU089289.1	4880315	4908757	tig00001019	1	28446	28443	99.89	translocation
MU089288.1	3655422	3683178	tig00009038	133058	105315	27757	99.94	reversion
MU089288.1	3696757	3723337	tig00009038	93666	67086	26581	100	reversion
MU089289.1	3145431	3171669	tig00001023	806043	832293	26239	99.46	translocation
MU089288.1	2948577	2974194	tig00001078	3795	29437	25618	99.89	translocation
JABTBS010000026.1	47775	72860	tig00001049	307668	332752	25086	99.92	translocation
MU089290.1	8283	32742	tig00001049	234799	210340	24460	99.98	reversion
JABTBS010000017.1	178001	201886	tig00000411	23902	1	23886	99.74	reversion
JABTBS010000012.1	3578798	3602631	tig00001046	230135	253968	23834	100	translocation

MU089290.1	102406	125600	tig00001049	185216	208411	23195	99.97	translocation
MU089288.1	2993265	3016045	tig00001078	168072	145291	22781	99.98	reversion
JABTBS010000012.1	3485181	3507479	tig00000052	1	22292	22299	99.97	translocation
JABTBS010000014.1	4883	26687	tig00001069	339	22090	21805	99.75	translocation
JABTBS010000012.1	3409686	3431395	tig00001052	824910	803175	21710	99.88	reversion
MU089294.1	80983	102652	tig00009033	21639	1	21670	99.84	reversion
MU089293.1	3470	25136	tig00009033	1	21639	21667	99.85	translocation
MU089293.1	157172	178838	tig00009033	21639	1	21667	99.85	reversion
JABTBS010000050.1	5377	27043	tig00009033	1	21639	21667	99.85	translocation
JABTBS010000050.1	44672	66337	tig00009033	1	21639	21666	99.85	translocation
MU089294.1	2387	24044	tig00009033	21639	1	21658	99.81	reversion
MU089294.1	96726	118373	tig00009033	21615	1	21648	99.83	reversion
MU089293.1	117902	139544	tig00009033	21639	1	21643	99.6	reversion
MU089294.1	33839	55481	tig00009033	21615	1	21643	99.84	reversion
MU089293.1	125762	147403	tig00009033	21615	1	21642	99.86	reversion
MU089293.1	133621	155262	tig00009033	21615	1	21642	99.86	reversion
MU089293.1	165056	186697	tig00009033	21615	1	21642	99.86	reversion
MU089294.1	10262	31903	tig00009033	21615	1	21642	99.86	reversion
MU089294.1	41699	63340	tig00009033	21615	1	21642	99.86	reversion
JABTBS010000051.1	7999	29640	tig00009033	21615	1	21642	99.82	reversion
JABTBS010000051.1	15858	37499	tig00009033	21615	1	21642	99.86	reversion
JABTBS010000056.1	1622	23249	tig00009033	1	21639	21628	99.64	translocation
JABTBS010000050.1	52530	73967	tig00009033	1	21453	21438	99.92	translocation
MU089294.1	104563	125835	tig00009033	21639	398	21273	99.84	reversion
MU089289.1	540237	561089	tig00000079	540947	520137	20853	99.8	reversion
JABTBS010000051.1	104	20847	tig00009033	21639	935	20744	99.67	reversion
MU089294.1	25955	46519	tig00009033	21639	1104	20565	99.83	reversion
JABTBS010000014.1	176726	196947	tig00001070	157475	177696	20222	100	translocation
JABTBS010000014.1	558681	578695	tig00001038	205772	225786	20015	99.88	translocation
JABTBS010000047.1	8882	28694	tig00001049	267869	287678	19813	99.67	translocation
JABTBS010000012.1	976503	996214	tig00000174	80120	99831	19712	99.99	translocation
JABTBS010000014.1	177303	196947	tig00000162	1	19651	19645	99.96	translocation
JABTBS010000014.1	1192063	1211700	tig00001040	1337116	1356753	19638	99.99	translocation
JABTBS010000056.1	11815	31108	tig00009033	2376	21639	19294	99.81	translocation
JABTBS010000050.1	1	19184	tig00009033	2484	21639	19184	99.83	translocation
JABTBS010000051.1	3927	23048	tig00001112	4529	23688	19122	99.03	translocation
MU089294.1	92661	111781	tig00001112	4529	23688	19121	99.17	translocation
MU089294.1	84802	103919	tig00001112	4529	23688	19118	99.18	translocation
JABTBS010000051.1	43229	62346	tig00001112	4529	23688	19118	99.18	translocation
MU089294.1	21915	41030	tig00001112	4529	23688	19116	99.18	translocation
MU089294.1	29774	48889	tig00001112	4529	23688	19116	99.18	translocation
JABTBS010000051.1	35370	54485	tig00001112	4529	23688	19116	99.19	translocation
MU089293.1	2203	21317	tig00001112	23688	4529	19115	99.2	reversion
MU089293.1	10062	29176	tig00001112	23688	4529	19115	99.2	reversion

MU089293.1	17921	37035	tig00001112	23688	4529	19115	99.2	reversion
MU089293.1	25780	44894	tig00001112	23688	4529	19115	99.2	reversion
MU089293.1	33639	52753	tig00001112	23688	4529	19115	99.2	reversion
MU089293.1	41498	60612	tig00001112	23688	4529	19115	99.2	reversion
MU089293.1	49357	68471	tig00001112	23688	4529	19115	99.2	reversion
MU089293.1	57216	76330	tig00001112	23688	4529	19115	99.2	reversion
MU089293.1	65075	84189	tig00001112	23688	4529	19115	99.2	reversion
MU089293.1	72934	92048	tig00001112	23688	4529	19115	99.2	reversion
MU089293.1	80793	99907	tig00001112	23688	4529	19115	99.2	reversion
MU089293.1	88652	107766	tig00001112	23688	4529	19115	99.2	reversion
MU089293.1	121697	140811	tig00001112	4529	23688	19115	99.19	translocation
MU089293.1	129556	148670	tig00001112	4529	23688	19115	99.2	translocation
MU089293.1	168850	187964	tig00001112	4529	23688	19115	99.2	translocation
MU089294.1	6197	25311	tig00001112	4529	23688	19115	99.2	translocation
MU089294.1	14056	33170	tig00001112	4529	23688	19115	99.2	translocation
MU089294.1	37634	56748	tig00001112	4529	23688	19115	99.2	translocation
MU089294.1	45493	64607	tig00001112	4529	23688	19115	99.2	translocation
MU089294.1	53352	72466	tig00001112	4529	23688	19115	99.2	translocation
JABTBS010000051.1	11793	30907	tig00001112	4529	23688	19115	99.2	translocation
JABTBS010000051.1	19652	38766	tig00001112	4529	23688	19115	99.2	translocation
JABTBS010000050.1	4110	23224	tig00001112	23688	4529	19115	99.2	reversion
JABTBS010000050.1	11969	31083	tig00001112	23688	4529	19115	99.2	reversion
JABTBS010000050.1	27687	46801	tig00001112	23688	4529	19115	99.2	reversion
JABTBS010000050.1	51263	70377	tig00001112	23688	4529	19115	99.2	reversion
JABTBS010000056.1	23893	43007	tig00001112	23688	4529	19115	99.2	reversion
MU089293.1	137415	156528	tig00001112	4529	23688	19114	99.19	translocation
MU089293.1	153133	172246	tig00001112	4529	23688	19114	99.19	translocation
JABTBS010000050.1	43405	62518	tig00001112	23688	4529	19114	99.19	reversion
JABTBS010000056.1	356	19430	tig00001112	23688	4529	19075	98.95	reversion
MU089289.1	848751	867645	tig00000079	192189	173301	18895	99.9	reversion
JABTBS010000021.1	22066	40887	tig00001087	1017400	1036210	18822	97.08	translocation
MU089289.1	531678	550417	tig00000079	549505	530767	18740	99.99	reversion
MU089294.1	77490	96057	tig00001112	5061	23688	18568	98.94	translocation
JABTBS010000015.1	2940207	2958553	tig00001083	757296	738950	18347	99.99	reversion
MU089293.1	96511	114736	tig00001112	23688	5416	18226	99.15	reversion
JABTBS010000017.1	160055	178003	tig00000411	43700	25762	17949	99.94	reversion
JABTBS010000014.1	338196	355656	tig00001068	34051	51532	17461	99.88	translocation
JABTBS010000011.1	1	17457	tig00009033	4210	21639	17457	99.81	translocation
MU089294.1	108382	125835	tig00001112	4529	22001	17454	99.42	translocation
MU089294.1	1	17452	tig00001112	6184	23688	17452	99.08	translocation
JABTBS010000051.1	43686	61078	tig00009033	17391	1	17393	99.99	reversion
JABTBS010000012.1	3512883	3530242	tig00000052	22292	39617	17360	99.75	translocation
MU089294.1	77490	94790	tig00009033	17317	1	17301	99.73	reversion
MU089293.1	1	17277	tig00009033	4387	21639	17277	99.79	translocation

MU089293.1	153864	170979	tig00009033	17117	1	17116	99.99	reversion
JABTBS010000012.1	2688358	2705313	tig00009047	1	16956	16956	100	translocation
JABTBS010000018.1	1438166	1454875	tig00000260	148500	131810	16710	99.03	reversion
JABTBS010000018.1	683522	700213	tig00000270	613154	629863	16692	98.98	translocation
JABTBS010000023.1	97716	114146	tig00000322	155249	138798	16431	99.59	reversion
JABTBS010000026.1	200570	216706	tig00001049	18027	34163	16137	99.99	translocation
JABTBS010000026.1	184450	200573	tig00001049	1	16101	16124	99.86	translocation
JABTBS010000018.1	16032	32056	tig00000260	745538	761562	16025	100	translocation
JABTBS010000014.1	6466	22350	tig00001068	154739	170619	15885	99.91	translocation
JABTBS010000059.1	3159	18981	tig00009033	21639	5845	15823	99.75	reversion
MU089294.1	1	15779	tig00009033	16194	407	15779	99.94	reversion
JABTBS010000026.1	182490	198231	tig00001071	15739	1	15742	99.97	reversion
MU089293.1	117216	132952	tig00001112	7883	23688	15737	98.58	translocation
JABTBS010000006.1	198914	214586	tig00001101	19813	4121	15673	99.83	reversion
JABTBS010000012.1	3343329	3358982	tig00000071	15678	1	15654	99.04	reversion
JABTBS010000056.1	11815	27289	tig00001112	20012	4529	15475	99.55	reversion
JABTBS010000017.1	95706	111165	tig00000423	145654	130223	15460	99.62	reversion
JABTBS010000056.1	1	15390	tig00009033	6238	21639	15390	99.48	translocation
JABTBS010000014.1	691627	707008	tig00001038	90999	75588	15382	99.79	reversion
JABTBS010000006.1	199229	214586	tig00000381	218711	203334	15358	99.87	reversion
JABTBS010000050.1	8	15365	tig00001112	19892	4529	15358	99.6	reversion
MU089289.1	4388910	4404265	tig00001019	862231	877639	15356	99.66	translocation
MU089288.1	2429000	2444350	tig00001062	268994	253654	15351	99.93	reversion
JABTBS010000017.1	141263	156578	tig00000423	135601	120286	15316	100	reversion
MU089289.1	2396793	2412084	tig00000270	18953	3700	15292	94.66	reversion
JABTBS010000018.1	864547	879800	tig00001034	880746	865455	15254	94.65	reversion
JABTBS010000051.1	1	15189	tig00001112	8469	23688	15189	98.73	translocation
MU089289.1	1506279	1521421	tig00001019	1362447	1377592	15143	96.09	translocation
MU089290.1	180455	195565	tig00000240	75371	60261	15111	100	reversion
JABTBS010000015.1	2018541	2033547	tig00001058	1	14932	15007	98.18	translocation
JABTBS010000015.1	1909335	1924057	tig00001087	231274	216500	14723	94.74	reversion
MU089293.1	117216	131685	tig00009033	14495	1	14470	99.5	reversion
MU089291.1	64416	78884	tig00001065	75111	89572	14469	99.59	translocation
MU089291.1	190144	204612	tig00001065	89572	75111	14469	99.59	reversion
JABTBS010000015.1	720391	734767	tig00001082	1	14409	14377	98.23	translocation
JABTBS010000013.1	88765	103015	tig00001103	111540	97301	14251	99.91	reversion
MU089289.1	1990224	2004462	tig00001034	577692	563438	14239	99.89	reversion
JABTBS010000015.1	2018541	2032755	tig00001057	1114630	1128780	14215	98.09	translocation
MU089293.1	176709	190734	tig00001112	4529	18551	14026	99.89	translocation
JABTBS010000015.1	719815	733803	tig00001081	176428	190468	13989	98.02	translocation
JABTBS010000012.1	2710054	2723993	tig00001052	78994	65073	13940	96.68	reversion
JABTBS010000051.1	1	13922	tig00009033	13909	1	13922	99.71	reversion
JABTBS010000006.1	4344	18156	tig00001106	1	13763	13813	98.22	translocation
JABTBS010000042.1	4368	18177	tig00001106	1	13760	13810	98.22	translocation

JABTBS010000045.1	16567	30376	tig00001106	1	13760	13810	98.22	translocation
JABTBS010000041.1	93455	107264	tig00001106	13760	1	13810	98.2	reversion
MU089286.1	3227770	3241578	tig00001106	13760	1	13809	98.2	reversion
JABTBS010000055.1	5658	19466	tig00001106	1	13760	13809	98.22	translocation
MU089289.1	5015565	5029373	tig00001106	13760	1	13809	98.2	reversion
JABTBS010000041.1	76022	89830	tig00001106	13760	1	13809	98.19	reversion
JABTBS010000032.1	89805	103613	tig00001106	13760	1	13809	98.19	reversion
JABTBS010000011.1	10243	24049	tig00001112	23688	9837	13807	98.89	reversion
JABTBS010000045.1	47808	61542	tig00001106	75	13760	13735	98.22	translocation
JABTBS010000045.1	61540	75273	tig00001106	75	13759	13734	98.22	translocation
JABTBS010000015.1	700727	714411	tig00001081	162748	176428	13685	98.32	translocation
JABTBS010000015.1	2004861	2018541	tig00001057	1095543	1109226	13681	98.39	translocation
JABTBS010000011.1	1	13638	tig00001112	18163	4529	13638	99.93	reversion
MU089291.1	65366	78885	tig00001066	1	13507	13520	99.54	translocation
MU089289.1	164675	178181	tig00000079	1273743	1260237	13507	99.62	reversion
MU089289.1	1135319	1148825	tig00000079	969834	983340	13507	99.62	translocation
MU089289.1	38456	51947	tig00001078	295120	281563	13492	99.51	reversion
MU089293.1	1	13458	tig00001112	17986	4529	13458	99.93	reversion
JABTBS010000015.1	1619159	1632556	tig00000083	15969	29360	13398	99.96	translocation
JABTBS010000018.1	1424778	1438170	tig00000260	162102	148707	13393	98.45	reversion
JABTBS010000018.1	669923	683315	tig00000270	599765	613158	13393	98.53	translocation
JABTBS010000012.1	3146426	3159783	tig00001052	518928	505571	13358	100	reversion
JABTBS010000014.1	176726	189998	tig00000174	13268	1	13273	99.92	reversion
MU089292.1	26975	40156	tig00000161	2785	15929	13182	99.53	translocation
MU089287.1	402020	415197	tig00000161	15929	2785	13178	99.51	reversion
MU089289.1	2833611	2846709	tig00000270	216160	229210	13099	95.61	translocation
JABTBS010000050.1	60945	73990	tig00009033	557	13616	13046	99.85	translocation
JABTBS010000050.1	60962	73990	tig00001112	21821	8762	13029	99.14	reversion
JABTBS010000045.1	1	12943	tig00001106	869	13760	12943	98.13	translocation
MU089292.1	33101	46026	tig00000319	211388	198464	12926	99.98	reversion
MU089287.1	396150	409074	tig00000319	198464	211388	12925	99.98	translocation
JABTBS010000039.1	42508	55328	tig00000319	211284	198464	12821	99.98	reversion
MU089289.1	2902080	2914846	tig00001034	822675	835426	12767	96.34	translocation
MU089289.1	2354013	2366763	tig00001025	64901	52134	12751	96.32	reversion
JABTBS010000041.1	25598	38317	tig00001106	12670	1	12720	98.06	reversion
MU089292.1	33481	46027	tig00000418	3	12562	12547	99.73	translocation
JABTBS010000039.1	22309	34855	tig00000418	3	12562	12547	99.73	translocation
MU089287.1	396149	408694	tig00000418	12562	3	12546	99.72	reversion
JABTBS010000041.1	41934	54434	tig00001106	13760	1321	12501	98.12	reversion
JABTBS010000012.1	358597	371074	tig00000174	688449	700926	12478	100	translocation
MU089289.1	2253297	2265707	tig00001025	167019	154635	12411	94.61	reversion
MU089289.1	2800026	2812411	tig00001034	721959	734369	12386	94.61	translocation
MU089290.1	1393885	1406028	tig00001049	272236	284379	12144	100	translocation
JABTBS010000026.1	13868	26011	tig00001049	272236	284379	12144	100	translocation

JABTBS010000059.1	6976	18981	tig00001112	4529	16532	12006	99.97	translocation
JABTBS010000012.1	2688064	2700041	tig00001084	21164	33134	11978	99.91	translocation
MU089289.1	3151746	3163577	tig00000050	11836	1	11832	99.25	reversion
JABTBS010000015.1	2120038	2131830	tig00001083	96891	85099	11793	99.99	reversion
JABTBS010000015.1	1786151	1797860	tig00001061	60751	49137	11710	95.53	reversion
MU089289.1	4018713	4030383	tig00001053	1	11676	11671	99.7	translocation
JABTBS010000015.1	878323	889937	tig00001061	966461	954752	11615	95.53	reversion
JABTBS010000056.1	1	11610	tig00001112	16139	4529	11610	99.97	reversion
JABTBS010000033.1	53311	64843	tig00001049	362579	351055	11533	99.63	reversion
JABTBS010000012.1	2684815	2696233	tig00001050	1048768	1037342	11419	99.76	reversion
MU089288.1	4006698	4018113	tig00000254	6536	17950	11416	99.98	translocation
JABTBS010000012.1	2618990	2630345	tig00001052	73242	61889	11356	99.96	reversion
JABTBS010000012.1	2684880	2696233	tig00001051	14589	3234	11354	99.96	reversion
JABTBS010000012.1	2712416	2723699	tig00009047	11282	1	11284	98.25	reversion
JABTBS010000051.1	51088	62356	tig00001112	4529	15795	11269	99.96	translocation
JABTBS010000017.1	531943	543192	tig00000123	24700	35967	11250	99.84	translocation
MU089292.1	54295	65505	tig00000319	211388	200119	11211	99.3	reversion
JABTBS010000041.1	84334	95518	tig00000517	7915	19090	11185	99.92	translocation
JABTBS010000042.1	33548	44732	tig00000517	19090	7916	11185	99.89	reversion
JABTBS010000042.1	33549	44732	tig00000020	20599	31759	11184	99.72	translocation
JABTBS010000041.1	84334	95517	tig00000020	31760	20599	11184	99.75	reversion
JABTBS010000045.1	10880	22063	tig00000517	19090	7915	11184	99.91	reversion
JABTBS010000045.1	10881	22063	tig00000020	20599	31760	11183	99.74	translocation
MU089289.1	5023877	5035057	tig00000517	7915	19090	11181	99.88	translocation
MU089289.1	5023877	5035056	tig00000020	31760	20599	11180	99.71	reversion
JABTBS010000006.1	1763152	1774331	tig00000086	1354099	1342931	11180	99.47	reversion
JABTBS010000015.1	740502	751526	tig00001083	3035	14051	11025	98.62	translocation
JABTBS010000015.1	2039716	2050733	tig00001059	12659	23683	11018	98.55	translocation
MU089288.1	4007109	4018113	tig00001075	1	11003	11005	99.94	translocation
JABTBS010000015.1	2502140	2513129	tig00001082	19456	30357	10990	91.41	translocation
JABTBS010000015.1	2038594	2049501	tig00001083	461304	472293	10908	91.28	translocation
MU089292.1	54675	65505	tig00000418	3	10906	10831	98.98	translocation
MU089289.1	880648	891451	tig00000079	154239	143435	10804	99.52	reversion
MU089294.1	63995	74785	tig00009033	15064	4273	10791	99.99	reversion
MU089287.1	9543	20292	tig00000260	273051	262360	10750	93.17	reversion
MU089293.1	103987	114736	tig00009033	6210	16962	10750	99.97	translocation
MU089289.1	2891278	2901903	tig00001034	812097	822664	10626	95.67	translocation
JABTBS010000012.1	3317533	3328153	tig00001050	1034592	1045203	10621	99.89	translocation
JABTBS010000012.1	3317533	3328153	tig00001051	484	11096	10621	99.9	translocation
MU089290.1	1332631	1343245	tig00001083	851168	840533	10615	98.82	reversion
MU089290.1	169850	180463	tig00000240	90622	80009	10614	100	reversion
JABTBS010000012.1	2616240	2626852	tig00001052	675854	686472	10613	99.9	translocation
MU089289.1	2382308	2392879	tig00001025	188316	198882	10572	96.17	translocation
MU089289.1	2343434	2354002	tig00001025	75703	65078	10569	95.67	reversion

MU089289.1	2768163	2778729	tig00001034	861541	850971	10567	96.18	reversion
JABTBS010000012.1	3044678	3055235	tig00001057	732601	722069	10558	99.75	reversion
MU089291.1	269355	279900	tig00001065	18073	7510	10546	99.71	reversion
JABTBS010000051.1	51819	62356	tig00009033	17117	6583	10538	99.97	reversion
MU089289.1	2742806	2753331	tig00000270	1154	11611	10526	93.45	translocation
JABTBS010000012.1	4720684	4731205	tig00001046	77904	67397	10522	99.05	reversion
MU089287.1	257751	268215	tig00001092	566245	555806	10465	98.73	reversion
MU089287.1	138277	148716	tig00001092	685741	675277	10440	98.73	reversion
JABTBS010000015.1	1307597	1317977	tig00001087	1008311	1018708	10381	92.52	translocation
MU089292.1	6372	16650	tig00000319	211388	201134	10279	99.68	reversion
MU089289.1	2424960	2435237	tig00001034	1130549	1140810	10278	96.68	translocation
MU089289.1	2658314	2668576	tig00001034	893623	903900	10263	96.69	translocation
JABTBS010000015.1	3094352	3104593	tig00001052	813076	823343	10242	99.75	translocation
JABTBS010000012.1	4720684	4730871	tig00001076	189047	178869	10188	98.97	reversion
JABTBS010000012.1	4720683	4730870	tig00009038	516722	526897	10188	99.02	translocation
JABTBS010000012.1	4720684	4730870	tig00000162	234270	244446	10187	99.25	translocation
JABTBS010000049.1	59382	69566	tig00001087	998879	988728	10185	99.58	reversion
MU089288.1	4384819	4394996	tig00000162	234270	244447	10178	99.53	translocation
JABTBS010000012.1	1292149	1302326	tig00001046	77905	67742	10178	99.47	reversion
MU089288.1	3475080	3485256	tig00001046	77904	67743	10177	99.21	reversion
MU089288.1	4384819	4394995	tig00001076	189047	178870	10177	99.83	reversion
MU089288.1	3475080	3485256	tig00009038	516723	526897	10177	99.89	translocation
MU089289.1	4020235	4030403	tig00001053	1526	11676	10169	99.19	translocation
JABTBS010000012.1	3751830	3761992	tig00000162	234269	244447	10163	99.45	translocation
JABTBS010000052.1	30663	40825	tig00001023	433005	443137	10163	99.7	translocation
MU089293.1	22833	32995	tig00009033	11505	21639	10163	99.69	translocation
MU089293.1	30692	40854	tig00009033	11505	21639	10163	99.69	translocation
MU089293.1	38551	48713	tig00009033	11505	21639	10163	99.69	translocation
MU089293.1	46410	56572	tig00009033	11505	21639	10163	99.69	translocation
MU089293.1	54269	64431	tig00009033	11505	21639	10163	99.69	translocation
MU089293.1	62128	72290	tig00009033	11505	21639	10163	99.69	translocation
MU089293.1	69987	80149	tig00009033	11505	21639	10163	99.69	translocation
MU089293.1	77846	88008	tig00009033	11505	21639	10163	99.69	translocation
MU089293.1	141455	151617	tig00009033	21639	11505	10163	99.69	reversion
JABTBS010000012.1	3751831	3761991	tig00001076	189047	178870	10161	99.17	reversion
JABTBS010000013.1	1534229	1544386	tig00009036	438426	428299	10158	99.7	reversion
JABTBS010000012.1	2753403	2763554	tig00001052	84266	94397	10152	99.34	translocation
JABTBS010000039.1	42508	52658	tig00000319	211307	201134	10151	99.74	reversion
JABTBS010000012.1	2753403	2763553	tig00009047	18566	8436	10151	99.34	reversion
JABTBS010000006.1	5180468	5190614	tig00001064	13060	23185	10147	99.24	translocation
MU089289.1	1570295	1580438	tig00001023	742325	732147	10144	98.96	reversion
JABTBS010000014.1	429384	439524	tig00001038	230748	220628	10141	99.34	reversion
JABTBS010000012.1	1081480	1091618	tig00000162	17843	27961	10139	99.8	translocation
MU089288.1	181435	191563	tig00001023	636728	626570	10129	99.7	reversion

JABTBS010000012.1	4133510	4143633	tig00000109	14396	4303	10124	99.68	reversion
JABTBS010000014.1	687000	697119	tig00001038	101147	90998	10120	99.7	reversion
JABTBS010000015.1	740502	750619	tig00001082	20578	30690	10118	98.71	translocation
MU089288.1	2692441	2702551	tig00001083	962624	972683	10111	99.49	translocation
JABTBS010000012.1	2583985	2594085	tig00001057	631517	621417	10101	99.96	reversion
MU089286.1	10201	20276	tig00000161	8829	18875	10076	99.47	translocation
JABTBS010000015.1	3094352	3104423	tig00001052	813076	823173	10072	99.74	translocation
JABTBS010000012.1	3066742	3076779	tig00001052	420018	430055	10038	99.99	translocation
JABTBS010000012.1	3066742	3076779	tig00001052	420018	430069	10038	99.85	translocation
JABTBS010000015.1	704371	714408	tig00001087	145942	135898	10038	95.23	reversion
MU089289.1	2306891	2316916	tig00000270	234819	244822	10026	96.49	translocation
MU089288.1	2761294	2771319	tig00001078	38364	28338	10026	99.89	reversion
JABTBS010000018.1	1092253	1102257	tig00001034	775556	785581	10005	96.47	translocation

Table S2. EF-1a sequences of 26 FOC1 isolates blast to NCBI

	Description	per ident	e-value	accession
FoYZ-1	Select seq MK172058.1 Fusarium oxysporum isolate FR3 translation elongation factor 1-alpha gene, partial cds	99.61	0	MK172058.1
FoYZ-2	Select seq MK172058.1 Fusarium oxysporum isolate FR3 translation elongation factor 1-alpha gene, partial cds	99.72	0	MK172058.1
FoYZ-3	Select seq MK172058.1 Fusarium oxysporum isolate FR3 translation elongation factor 1-alpha gene, partial cds	99.86	0	MK172058.1
FoYQ-1	Select seq MK172058.1 Fusarium oxysporum isolate FR3 translation elongation factor 1-alpha gene, partial cds	99.72	0	MK172058.1
FoYQ-2	Select seq MK172058.1 Fusarium oxysporum isolate FR3 translation elongation factor 1-alpha gene, partial cds	99.61	0	MK172058.1
FoXT-1	Select seq MK172058.1 Fusarium oxysporum isolate FR3 translation elongation factor 1-alpha gene, partial cds	99.61	0	MK172058.1
FoXT-2	Select seq MK172058.1 Fusarium oxysporum isolate FR3 translation elongation factor 1-alpha gene, partial cds	99.72	0	MK172058.1
FoXA-1	Select seq MK172058.1 Fusarium oxysporum isolate FR3 translation elongation factor 1-alpha gene, partial cds	99.61	0	MK172058.1
FoXA-2	Select seq MK172058.1 Fusarium oxysporum isolate FR3 translation elongation factor 1-alpha gene, partial cds	99.61	0	MK172058.1
FoWN-1	Select seq MK172058.1 Fusarium oxysporum isolate FR3 translation elongation factor 1-alpha gene, partial cds	99.72	0	MK172058.1
FoWN-2	Select seq MK172058.1 Fusarium oxysporum isolate FR3 translation elongation factor 1-alpha gene, partial cds	99.61	0	MK172058.1
FoTY-1	Select seq MK172058.1 Fusarium oxysporum isolate FR3 translation elongation factor 1-alpha gene, partial cds	99.86	0	MK172058.1
FoTY-2	Select seq MK172058.1 Fusarium oxysporum isolate FR3 translation elongation factor 1-alpha gene, partial cds	99.61	0	MK172058.1
FoTY-	Select seq MK172058.1 Fusarium oxysporum isolate FR3 translation elongation factor	99.61	0	

3	1-alpha gene, partial cds			MK172058.1
FoJZ-1	Select seq MK172058.1 Fusarium oxysporum isolate FR3 translation elongation factor 1-alpha gene, partial cds	99.86	0	MK172058.1
FoJZ-2	Select seq MK172058.1 Fusarium oxysporum isolate FR3 translation elongation factor 1-alpha gene, partial cds	99.72	0	MK172058.1
FoHD-1	Select seq MK172058.1 Fusarium oxysporum isolate FR3 translation elongation factor 1-alpha gene, partial cds	99.61	0	MK172058.1
FoHD-2	Select seq MK172058.1 Fusarium oxysporum isolate FR3 translation elongation factor 1-alpha gene, partial cds	99.86	0	MK172058.1
FoDX-1	Select seq MK172058.1 Fusarium oxysporum isolate FR3 translation elongation factor 1-alpha gene, partial cds	99.72	0	MK172058.1
FoDX-2	Select seq MK172058.1 Fusarium oxysporum isolate FR3 translation elongation factor 1-alpha gene, partial cds	99.61	0	MK172058.1
FoDX-3	Select seq MK172058.1 Fusarium oxysporum isolate FR3 translation elongation factor 1-alpha gene, partial cds	99.86	0	MK172058.1
FoCP-1	Select seq MK172058.1 Fusarium oxysporum isolate FR3 translation elongation factor 1-alpha gene, partial cds	99.72	0	MK172058.1
FoCP-2	Select seq MK172058.1 Fusarium oxysporum isolate FR3 translation elongation factor 1-alpha gene, partial cds	99.72	0	MK172058.1
FoBD-1	Select seq MK172058.1 Fusarium oxysporum isolate FR3 translation elongation factor 1-alpha gene, partial cds	99.61	0	MK172058.1
FoBD-2	Select seq MK172058.1 Fusarium oxysporum isolate FR3 translation elongation factor 1-alpha gene, partial cds	99.61	0	MK172058.1
FoBD-3	Select seq MK172058.1 Fusarium oxysporum isolate FR3 translation elongation factor 1-alpha gene, partial cds	99.61	0	MK172058.1

Table S3. ITS sequences of 26 FOC1 isolates blast to NCBI

	Description	per ident	e-value	accession
FoYZ-1	Fusarium sp. P. del g internal transcribed spacer 1, partial sequence; 5.8S ribosomal RNA gene and internal transcribed spacer 2, complete sequence; and 28S ribosomal RNA gene, partial sequence	99.59	0	JX243754.1
FoYZ-2	Fusarium sp. P. del g internal transcribed spacer 1, partial sequence; 5.8S ribosomal RNA gene and internal transcribed spacer 2, complete sequence; and 29S ribosomal RNA gene, partial sequence	98.77	0	JX243754.1
FoYZ-3	Fusarium sp. P. del g internal transcribed spacer 1, partial sequence; 5.8S ribosomal RNA gene and internal transcribed spacer 2, complete sequence; and 30S ribosomal RNA gene, partial sequence	99.59	0	JX243754.1
FoYQ-1	Fusarium sp. P. del g internal transcribed spacer 1, partial sequence; 5.8S ribosomal RNA gene and internal transcribed spacer 2, complete sequence; and 31S ribosomal RNA gene, partial sequence	98.77	0	JX243754.1
FoYQ-2	Fusarium sp. P. del g internal transcribed spacer 1, partial sequence; 5.8S ribosomal RNA gene and internal transcribed spacer 2, complete sequence; and 32S ribosomal RNA gene, partial sequence	98.77	0	JX243754.1
FoXT-1	Fusarium sp. P. del g internal transcribed spacer 1, partial sequence; 5.8S ribosomal RNA gene and internal transcribed spacer 2, complete sequence; and 33S ribosomal RNA gene, partial sequence	99.18	0	JX243754.1
FoXT-2	Fusarium sp. P. del g internal transcribed spacer 1, partial sequence; 5.8S ribosomal RNA gene and internal transcribed spacer 2, complete sequence; and 34S ribosomal RNA gene, partial sequence	99.18	0	JX243754.1
FoXA-1	Fusarium sp. P. del g internal transcribed spacer 1, partial sequence; 5.8S ribosomal RNA gene and internal transcribed spacer 2, complete sequence; and 35S ribosomal RNA gene, partial sequence	99.59	0	JX243754.1
FoXA-2	Fusarium sp. P. del g internal transcribed spacer 1, partial sequence; 5.8S ribosomal RNA gene and internal transcribed spacer 2, complete sequence; and 36S ribosomal RNA gene, partial sequence	98.77	0	JX243754.1
FoWN-1	Fusarium sp. P. del g internal transcribed spacer 1, partial sequence; 5.8S ribosomal RNA gene and internal transcribed spacer 2, complete sequence; and 37S ribosomal RNA gene, partial sequence	98.77	0	JX243754.1
FoWN-2	Fusarium sp. P. del g internal transcribed spacer 1, partial sequence; 5.8S ribosomal RNA gene and internal transcribed spacer 2, complete sequence; and 38S ribosomal RNA gene, partial sequence	99.18	0	JX243754.1
FoTY-1	Fusarium sp. P. del g internal transcribed spacer 1, partial sequence; 5.8S ribosomal RNA gene and internal transcribed spacer 2, complete sequence; and 39S ribosomal RNA gene, partial sequence	98.77	0	JX243754.1
FoTY-2	Fusarium sp. P. del g internal transcribed spacer 1, partial sequence; 5.8S ribosomal RNA gene and internal transcribed spacer 2, complete sequence; and 40S ribosomal RNA gene, partial sequence	99.59	0	JX243754.

				1
FoTY-3	Fusarium sp. P. del g internal transcribed spacer 1, partial sequence; 5.8S ribosomal RNA gene and internal transcribed spacer 2, complete sequence; and 41S ribosomal RNA gene, partial sequence	99.18	0	JX243754.1
FoJZ-1	Fusarium sp. P. del g internal transcribed spacer 1, partial sequence; 5.8S ribosomal RNA gene and internal transcribed spacer 2, complete sequence; and 42S ribosomal RNA gene, partial sequence	99.59	0	JX243754.1
FoJZ-2	Fusarium sp. P. del g internal transcribed spacer 1, partial sequence; 5.8S ribosomal RNA gene and internal transcribed spacer 2, complete sequence; and 43S ribosomal RNA gene, partial sequence	99.59	0	JX243754.1
FoHD-1	Fusarium sp. P. del g internal transcribed spacer 1, partial sequence; 5.8S ribosomal RNA gene and internal transcribed spacer 2, complete sequence; and 44S ribosomal RNA gene, partial sequence	98.77	0	JX243754.1
FoHD-2	Fusarium sp. P. del g internal transcribed spacer 1, partial sequence; 5.8S ribosomal RNA gene and internal transcribed spacer 2, complete sequence; and 45S ribosomal RNA gene, partial sequence	99.18	0	JX243754.1
FoDX-1	Fusarium sp. P. del g internal transcribed spacer 1, partial sequence; 5.8S ribosomal RNA gene and internal transcribed spacer 2, complete sequence; and 46S ribosomal RNA gene, partial sequence	98.77	0	JX243754.1
FoDX-2	Fusarium sp. P. del g internal transcribed spacer 1, partial sequence; 5.8S ribosomal RNA gene and internal transcribed spacer 2, complete sequence; and 47S ribosomal RNA gene, partial sequence	98.77	0	JX243754.1
FoDX-3	Fusarium sp. P. del g internal transcribed spacer 1, partial sequence; 5.8S ribosomal RNA gene and internal transcribed spacer 2, complete sequence; and 48S ribosomal RNA gene, partial sequence	99.18	0	JX243754.1
FoCP-1	Fusarium sp. P. del g internal transcribed spacer 1, partial sequence; 5.8S ribosomal RNA gene and internal transcribed spacer 2, complete sequence; and 49S ribosomal RNA gene, partial sequence	99.18	0	JX243754.1
FoCP-2	Fusarium sp. P. del g internal transcribed spacer 1, partial sequence; 5.8S ribosomal RNA gene and internal transcribed spacer 2, complete sequence; and 50S ribosomal RNA gene, partial sequence	98.77	0	JX243754.1
FoBD-1	Fusarium sp. P. del g internal transcribed spacer 1, partial sequence; 5.8S ribosomal RNA gene and internal transcribed spacer 2, complete sequence; and 51S ribosomal RNA gene, partial sequence	99.18	0	JX243754.1
FoBD-2	Fusarium sp. P. del g internal transcribed spacer 1, partial sequence; 5.8S ribosomal RNA gene and internal transcribed spacer 2, complete sequence; and 52S ribosomal RNA gene, partial sequence	99.59	0	JX243754.1
FoBD-3	Fusarium sp. P. del g internal transcribed spacer 1, partial sequence; 5.8S ribosomal RNA gene and internal transcribed spacer 2, complete sequence; and 53S ribosomal RNA gene, partial sequence	99.59	0	JX243754.1

Table S4. the sequence of FOC-specific DNA fragment

id	sequence
tig00009029_548994_549339_+	AATTTGCTGTGATAGGTGGATTTACGGCGCAATATCGGAAAAGAGGTTAAATGCAAATTCTGCATCGTCAGCTGCGGCGGTAGTGGACGCCATGAGGGTGACGAAGAAAAGGCGAAAGCCCACGGTGGACTTCATATCGTTGAACAACATGGTTTGAAAACTAATATTGAGAAACTGATTTGGATAGAAAAGAGGTTCCATGGAAAACCAGGAGTGGCTTATGACAGCTTTTGTGATAATACAAAATCCCCCAGACCCCAACCGGTACCCATGTTACTTATCACCCTCCATGTCTTAACTAAAAAGTGCAGCACTCTAACACCGAAACCCTTGTCACTATCATTGA
